# CSF and Brain Structural Imaging Markers of the Alzheimer's Pathological Cascade

**DOI:** 10.1371/journal.pone.0047406

**Published:** 2012-12-19

**Authors:** Xianfeng Yang, Ming Zhen Tan, Anqi Qiu

**Affiliations:** 1 Department of Bioengineering, National University of Singapore, Singapore, Singapore; 2 Singapore Institute for Clinical Sciences, the Agency for Science, Technology and Research, Singapore, Singapore; 3 Clinical Imaging Research Center, National University of Singapore, Singapore, Singapore; Institution of Automation, CAS, China

## Abstract

Cerebral spinal fluid (CSF) and structural imaging markers are suggested as biomarkers amended to existing diagnostic criteria of mild cognitive impairment (MCI) and Alzheimer's disease (AD). But there is no clear instruction on which markers should be used at which stage of dementia. This study aimed to first investigate associations of the CSF markers as well as volumes and shapes of the hippocampus and lateral ventricles with MCI and AD at the baseline and secondly apply these baseline markers to predict MCI conversion in a two-year time using the Alzheimer's Disease Neuroimaging Initiative (ADNI) cohort. Our results suggested that the CSF markers, including Aβ42, t-tau, and p-tau, distinguished MCI or AD from NC, while the Aβ42 CSF marker contributed to the differentiation between MCI and AD. The hippocampal shapes performed better than the hippocampal volumes in classifying NC and MCI, NC and AD, as well as MCI and AD. Interestingly, the ventricular volumes were better than the ventricular shapes to distinguish MCI or AD from NC, while the ventricular shapes showed better accuracy than the ventricular volumes in classifying MCI and AD. As the CSF markers and the structural markers are complementary, the combination of them showed great improvements in the classification accuracies of MCI and AD. Moreover, the combination of these markers showed high sensitivity but low specificity for predicting conversion from MCI to AD in two years. Hence, it is feasible to employ a cross-sectional sample to investigate dynamic associations of the CSF and imaging markers with MCI and AD and to predict future MCI conversion. In particular, the volumetric information may be good for the early stage of AD, while morphological shapes should be considered as markers in the prediction of MCI conversion to AD together with the CSF markers.

## Introduction

Cerebral spinal fluid (CSF) and imaging markers have been suggested as biomarkers to augment existing diagnostic criteria of both mild cognitive impairment (MCI) and Alzheimer's disease (AD) [1], [2], [3], [4], [5], [6]. Jack et al. [7] proposed a possible hypothetical model in which biomarkers were temporally arranged in order of abnormality along the pathological cascade of AD. In this model, abnormal CSF Aβ42 could occur two decades before the first dementia-related symptoms, reaching a plateau prior any manifestation of cognitive impairment. In comparison to trajectories of CSF tau, magnetic resonance imaging (MRI) markers surfaced much later and were well correlated with the severity of AD symptoms. However, this hypothetical model was directly derived from longitudinal studies, where statistical inferences were founded primarily on the rate of change of AD-related biomarkers over time. For instance, increased rates of ventricular expansion and brain atrophy in the medial temporal lobe were found to be significantly correlated with cognitive decline, with good predictions for MCI to AD conversion [8] [9] [10].

Beyond simple volumetric measures, morphological shape of the brain captures not only the degree of tissue loss but also its precise anatomical location. As such, brain shape measures have since been suggested as improved predictors for MCI conversion to AD. For instance, changes of hippocampal shapes between baseline and a 2-year follow-up predicted MCI-AD conversion up to 80% accuracy [11], [12]. Unfortunately, to date, no clear, authoritative instruction on which structural MRI measures are to be associated with MCI and AD is available. This could be, partly, a result of the extensive variety of image analysis techniques available. In addition, while recent studies [13], [14] have tested the feasibility of baseline structural volumes and CSF in predicting conversion from MCI to AD, performance of baseline structural shapes with CSF markers for MCI-AD conversion remains relatively unknown.

In this paper, we first evaluated the hypothetical model suggested by Jack et al. [7] through a cross-sectional study on the Alzheimer's Disease Neuroimaging Initiative (ADNI) cohort. For this, we employed a supervised, multivariate classification method, support vector machine (SVM), to distinguish MCI and AD from normal aging. Features used include CSF biomarkers and the shapes and volumes of hippocampus and lateral ventricles. The hippocampi and lateral ventricles were chosen for their well-validated status as prominent hallmarks of AD [8], [15], [16], [17], [18], [19], [20]. Subsequently, we aim to predict MCI conversion to AD over a two-year follow-up period using baseline CSF and MRI measures. In particular, both volumetric and shape analyses were applied to compare their sensitivity and specificity in the prediction of MCI conversion to AD.

## Methods

The ADNI was launched in 2003 by the National Institute on Aging (NIA), the National Institute of Biomedical Imaging and Bioengineering (NIBIB), the Food and Drug Administration [21], private pharmaceutical companies and non-profit organizations, as a $60 million, 5-year public–private partnership. The primary goal of ADNI has been to test whether serial MRI, PET, other biological markers, and clinical and neuropsychological assessments can be combined to measure the progression of MCI and early AD. Determination of sensitive and specific markers of very early AD progression is intended to aid researchers and clinicians to develop new treatments and monitor their effectiveness, as well as lessen the time and cost of clinical trials.

ADNI is the result of efforts of many coinvestigators from a broad range of academic institutions and private corporations, and subjects have been recruited from over 50 sites across the U.S. and Canada. The initial goal of ADNI was to recruit 800 adults, ages 55 to 90, to participate in the research — approximately 200 cognitively normal older individuals to be followed for 3 years, 400 people with MCI to be followed for 3 years, and 200 people with early AD to be followed for 2years (see www.adni-info.org for up-to-date information). The data were analyzed anonymously, using publicly available secondary data from the ADNI study, therefore no ethics statement is required for this work.

### Subjects

The ADNI general eligibility criteria are described at www.adni- info.org. Briefly, subjects are between 55–90 years of age, having a study partner able to provide an independent evaluation of functioning. Specific psychoactive medications will be excluded. General inclusion/exclusion criteria are as follows: 1) healthy subjects: Mini- Mental State Examination (MMSE) scores between 24–30, a Clinical Dementia Rating (CDR) of 0, non-depressed, non-MCI, and nondemented; 2) MCI subjects: MMSE scores between 24–30, a memory complaint, having objective memory loss measured by education adjusted scores on Wechsler Memory Scale Logical Memory II, a CDR of 0.5, absence of significant levels of impairment in other cognitive domains, essentially preserved activities of daily living, and an absence of dementia; and 3) mild AD: MMSE scores between 20–26, CDR of 0.5 or 1.0, and meets the National Institute of Neurological and Communicative Disorders and Stroke and the Alzheimer's Disease and Related Disorders Association (NINCDS/ADRDA) criteria for probable AD.

In this study, 383 subjects were chosen from our previous study [22]. Within this group, 218 have both MRI and CSF baseline data (age: 74.4±7.2 years), with 72 normal controls (NC), 35 AD subjects, and 111 MCI patients. Amongst these 383 subjects, 25 subjects with MCI converted to AD within 24 months.

Structural MR scans were collected across a variety of scanners with protocols individualized for each scanner, as defined at www.loni.ucla.edu/ADNI/Research/Cores/index.shtml. The CSF Aβ42, t-tau and p-tau data were downloaded from the ADNI web site (www.loni.ucla.edu/ADNI).

### MRI analysis

**Figure 1:**
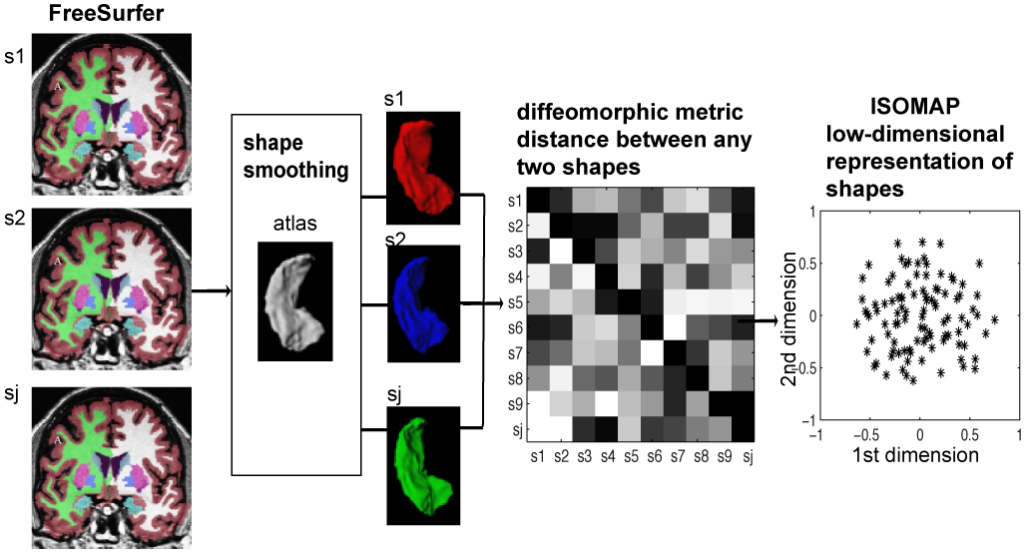
Schematic of MRI data processing.

Figure 1 illustrates the MRI data processing that is detailed below.

#### Structural Delineation

We automatically delineated the hippocampus (HC) and lateral ventricles (LV) from the intensity inhomogeneity corrected T1-weighted MR images using FreeSurfer [23]. Due to the lack of constraints on structural shapes, this process introduced irregularities and topological errors (e.g. holes) at the hippocampal and ventricular boundary. This would increase shape variation and thus reduces statistical power to detect group differences. To avoid this pitfall, we generated the hippocampal or ventricular shape of each individual subject with the properties of smoothness and correct topology by injecting an atlas shape into them using the large deformation diffeomorphic metric image mapping algorithm [24]. The hippocampal and lateral ventricular atlas shapes were created from 41 manually labeled hippocampi and lateral ventricles via a large deformation diffeomorphic atlas generation algorithm [25]. Each hippocampal (or lateral ventricular) volume was approximated by the transformed atlas through the LDDMM transformation. The reader is referred to [26] for the mathematical derivation of this atlas injection procedure and its evaluation as well as the segmentation accuracy on the hippocampus and lateral ventricles. This delineation approach has been successfully applied to investigate the hippocampus and other subcortical shapes in AD [22].

We constructed the surface representation of the hippocampus and lateral ventricles by composing the LDDMM transformation on the corresponding atlas surfaces [27]. The left and right hippocampal surfaces were respectively constructed using 2364 triangles with 1184 vertices and 2458 triangles with 1231 vertices, while the left and right lateral ventricular surfaces were respectively composed of 6966 triangles with 3485 vertices and 7890 triangles with 3947 vertices. The average triangle area and edge length of the hippocampal surfaces were respectively 0.59 mm^2^ and 1.2 mm, while those of the ventricular surfaces were respectively 0.64 mm^2^ and 1.1 mm. Hence, the size of the triangles was comparable to the image resolution (1 mm^3^).

#### ISOMAP Shape Embedding

Unlike the scalar volume measure, structural shapes lie on a high dimensional space, which makes it challenging for statistical inference. In this study, we employed ISOMAP [28] to embed the shapes of the hippocampus and lateral ventricles into a Euclidean space with a few dimensions such that this low-dimensional embedding is quasi-isometric to the shapes in the high dimensional space. For this, we first computed diffeomorphic metric distances between any two shapes using their first order approximation described in [29] and constructed a pair-wise distance matrix. ISOMAP then found a Euclidean low-dimensional representation of the shapes that preserved the relationship of any two shapes described in the pair-wise distance matrix. These Euclidean coordinates were obtained by finding eigenvectors corresponding to the largest eigenvalues of the kernel matrix stemmed from the distance matrix reshaped by a centering matrix [28]. The dimension of the eigenvectors is the same as the number of subjects, and each eigenvector is one component or one dimension of the ISOMAP shape embedding. Using this approach with all 383 subjects, the bilateral hippocampal shapes can be characterized using the first 20 ISOMAP components whose Euclidean distance matrix is highly correlated with the pair-wise metric distance matrix generated using the first order approximation of the diffeomorphic metric (Pearson's Correlation: r = 0.91). The bilateral lateral ventricular shapes can be represented using the first 20 ISOMAP components whose Euclidean distance matrix is very much similar to the pair-wise metric distance matrix (Pearson's Correlation: r = 0.97).

### Statistical Analysis

A linear support vector machine (SVM) [30] was employed to identify diagnosis of subjects from any two groups (NC, MCI, and AD). The SVM classifier seeks the optimal decision boundary that has a maximal margin closest to the training samples such that generalization error bound can be minimized. Hence, the SVM classifier is robust to outliers. In our study, we independently and jointly considered volumes (or shapes) of the hippocampus, lateral ventricles and CSF markers as the SVM input features to identify subjects with MCI and AD from NC and MCI from AD. Shape features comprise only of ISOMAP components with significant group differences based on Student *t*-tests.

Our study had fewer AD subjects as compared to the NC or MCI groups. To resolve any influences of unequal sample sizes among the NC, MCI, and AD groups on the classification accuracy, we employed random sampling to reduce the number of NC and MCI subjects such that all three groups have equal sample sizes. This was repeated for 100 times. For each trial, leave-one-out cross-validation was adopted to estimate the classification accuracy. The confidence interval of the classification accuracy was computed.

Moreover, we also applied the SVM to test the sensitivity and specificity for predicting MCI conversion in a two-year time window when volumes (or shapes) of the hippocampus and lateral ventricles as well as CSF markers assessed at the baseline were independently or jointly considered as features in the SVM.

## Results

### Demographic Information

Demographic information for different diagnosis groups at baseline are shown in **Table 1**. No significant differences in age were found among the NC, MCI, and AD groups (ANOVA, p = 0.454). 25 out of 111 MCI subjects were diagnosed as AD at the two-year follow up and were denoted as the MCI-c group. Rest of the MCI subjects were placed in the MCI-s group. No significant MMSE difference was found between the two MCI groups at baseline (p = 0.10).

**Table 1:** Demographic information for each of the diagnosis groups (normal controls (NC), mild cognitive impairment (MCI), and Alzheimer's disease (AD)) at the baseline.

Group	Subjects *n*	Age (mean±SD)	Gender (female/male)	MMSE (mean±SD)
NC	72	75.2±5.2	35/37	29±1
MCI-s	86	74±7.7	28/58	26.7±1.8
MCI-c	25	73.5±6.9	6/19	26.1±1.5
AD	35	74.6±9.3	15/20	22.9±1.8

Note: SD – standard deviation; MMSE – mini-mental state examination; MCI-s – subjects with MCI who remained as MCI at the two-year follow up; MCI-c – subjects with MCI who converted as AD at the two-year follow up.

### Markers at Stages of MCI an AD

#### Hippocampal volume and shape markers

Bilateral hippocampal volumes distinguished MCI and AD subjects from normal controls at an accuracy of 61.9% and 65.5% respectively, with relatively high specificity (MCI: 66.1%; AD: 73.3%) but low sensitivity (MCI: 57.7%; AD: 57.8%) (**Table 2**). However, the hippocampal volumes lost statistical power in the separation of subjects with MCI and AD (classification accuracy: 42.3%; sensitivity: 45.3%; specificity: 39.2%, **Table 2**).

**Table 2:** The classification accuracy, sensitivity, and specificity of the support vector machine (SVM) classifiers are given for distinguishing normal controls (NC) and subjects with Alzheimer's disease (AD), NC and subjects with mild cognitive impairment (MCI), and subjects with MCI and AD.

	NC.vs. AD	NC.vs. MCI	MCI.vs. AD
**Hippocampal Volumes and Shapes**			
Hp volumes	65.5% (CI: 64.4%∼66.6%) Sensitivity = 57.8%, Specificity = 73.3%	61.9% (CI: 61%∼62.8%) Sensitivity = 57.7%, Specificity = 66.1%	42.3% (CI:32.2%∼52.3%) Sensitivity = 45.3%, Specificity = 39.2%
Hp shapes	79.2% (CI: 78.5%∼80%) Sensitivity = 75.8%, Specificity = 82.8%	67.4% (CI: 66.2%∼68.6%) Sensitivity = 64%, Specificity = 70.8%	57.2% (CI: 51.7%∼62.8%) Sensitivity = 59.9%, Specificity = 54.5%
**Lateral Ventricular Volumes and Shapes**			
LV volumes	65.5% (CI: 64.9%∼66.1%) Sensitivity = 58.8% Specificity = 72.3%	63.1% (CI: 62%∼64.2%) Sensitivity = 50.9%, Specificity = 75.3%	30.4% (CI: 18.8%∼42%) Sensitivity = 21.2% Specificity = 39.7%
LV shapes	61.5% (CI: 59.2%∼63.9%) Sensitivity = 59.2%, Specificity = 63.9%	59% (CI: 56.1%∼62%) Sensitivity = 53.6%, Specificity = 64.4%	60.1% (CI:57.1%∼63.1%) Sensitivity = 62%, Specificity = 58.3%
**CSF**			
CSF markers	81.4% (CI: 80.3%∼82.5%) Sensitivity = 87.4%, Specificity = 74.9%	68.4% (CI: 67.8%∼69%) Sensitivity = 66.7%, Specificity = 70.1%	61.3% (CI: 59.4%∼63.2%) Sensitivity = 84.3%, specificity = 38.3%
**Combination**			
CSF, Hp volumes, LV volumes	85.4% (CI: 84.3%∼86.5%) Sensitivity = 88.8%, Specificity = 82%	72% (CI: 70.5%∼73.5%) Sensitivity = 70.1%, Specificity = 73.9%	60.9% (CI: 59.1%∼62.8%) Sensitivity = 80.4%, Specificity = 41.4%
CSF, Hp shapes, LV shapes	92.2% (CI: 91%∼93.5%) Sensitivity = 94.7%, Specificity = 89.8%	70.3% (CI: 68.6%∼72.9%) Sensitivity = 69.5%, specificity = 71.9%	69.6% (CI: 66.4%∼72.8%) Sensitivity = 70.7%, Specificity = 68.6%

The volumes and shapes of the hippocampus (Hp) and lateral ventricles (LV) as well as cerebral spinal fluid (CSF) markers are respectively used as features in the SVM.

**Figure 2:**
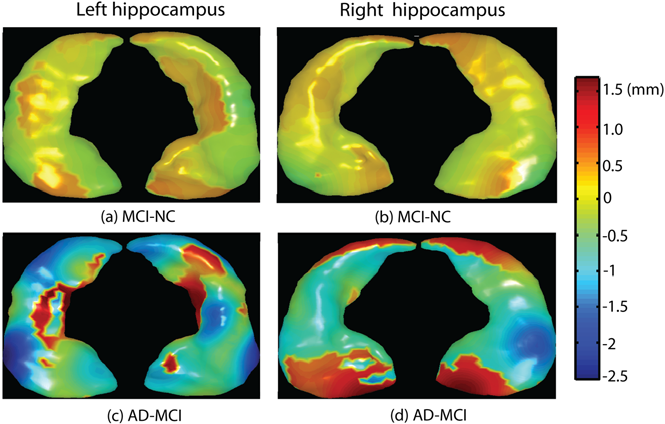
Hippocampal shape differences among normal controls (NC), mild cognitive impairment (MCI), and Alzheimer's Disease (AD). Panels (a,b) respectively show group differences in the left and right hippocampal surface deformations between MCI and NC. Panels (c,d) respectively show group differences in the left and right hippocampal surface deformations between AD and MCI. Warm color denotes regions where structures have surface outward-deformation in the former group when compared with the latter group, while cool color denotes regions where structures have surface inward-deformation in the former group when compared with the latter group.

In shape analysis, the first 20 ISOMAP components characterized bilateral hippocampal shapes among all 383 subjects. The 1^st^, 2^nd^, 3^rd^, 4^th^, 9^th^, 13^th^, 18^th^ components contributed to hippocampal shape differences between NC and AD. Among these components, most of them (the 1^st^, 2^nd^, 3^rd^, 7^th^, 13^th^) also contributed to shape differences between NC and MCI (**Table 3**). Interestingly, only the 4^th^ and 9^th^ components showed shape differences between MCI and AD. Moreover, among these ISOMAP components, the 1^st^ and 2^nd^ components were highly correlated with the hippocampal volume (Pearson correlations: r = 0.7, p<0.01 for the 1^st^ component; r = 0.5, p<0.01 for the 2^nd^ component), which were dominant components for group differences in hippocampal shapes between NC and MCI. This is illustrated as relatively homogeneous shrinkage over the bilateral hippocampi in **Figure 2 (a,b)**. But the 4^th^ and 9^th^ components were not associated with the hippocampal volume (p>0.05), suggesting that only local hippocampal shapes contributed to the difference between MCI and AD, as seen in **Figure 2 (c,d)**. This can be further supported by evidence of increased classification accuracy rates for the classifications between NC and AD (79.2%), NC and MCI (67.4%), and MCI and AD (57.2%) (**Table 2**) when the ISOMAP embedding of hippocampal shapes were used in the SVM classifiers.

**Table 3:** ISOMAP components of the hippocampus and lateral ventricles as well as CSF markers contribute to the group differences between normal controls (NC) and subjects with Alzheimer's disease (AD), NC and subjects with mild cognitive impairment (MCI), and subjects with MCI and AD.

	NC.vs. AD	NC.vs. MCI	MCI vs AD
**Imaging Markers**			
	ISOMAP components	ISOMAP components	ISOMAP components
**hippocampal shapes**	1,2,3,4,9,13,18	1,2,3,7,13	4,9
**lateral ventricular shapes**	1,7,8,9,17,19	1,8,12,14	9, 13,17
**CSF Markers**			
**CSF Aβ_42_, t-tau, p-tau**	Aβ42, t-tau, p-tau	Aβ42, t-tau, p-tau	Aβ42

#### Lateral ventricular volume and shape markers

The volumes of bilateral lateral ventricles distinguished subjects with MCI and AD from normal controls at the accuracy of 63.1% and 65.5% respectively, with relatively high specificity (MCI: 75.3%; AD: 72.3%) but low sensitivity (MCI: 50.9%; AD: 58.8%) (**Table 2**). However, volumes of the lateral ventricles lost statistical power in separating subjects with MCI and AD (classification accuracy: 30.4%; sensitivity: 21.2%; specificity: 39.7%, **Table 2**).

**Figure 3:**
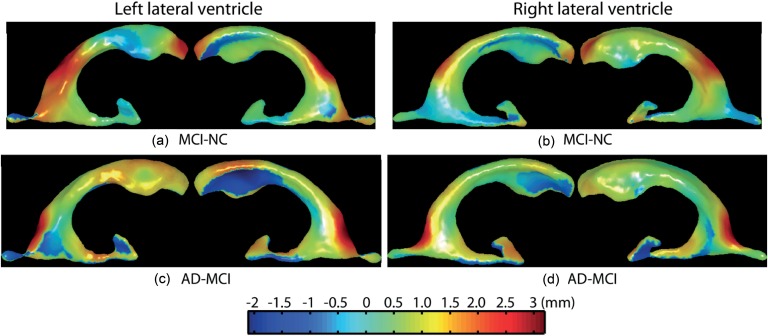
Shape differences of the lateral ventricles among normal controls (NC), mild cognitive impairment (MCI), and Alzheimer's Disease (AD). Panels (a,b) respectively show group differences in the left and right lateral ventricular surface deformations between MCI and NC. Panels (c,d) respectively show group differences in the left and right lateral ventricular surface deformations between AD and MCI. Warm color denotes regions where structures have surface outward-deformation in the former group when compared with the latter group, while cool color denotes regions where structures have surface inward-deformation in the former group when compared with the latter group.

In shape analysis, the first 20 ISOMAP components characterized bilateral lateral ventricular shapes among all 383 subjects. The 1^st^, 7^th^, 8^th^, 9^th^, 17^th^, 19^th^ components contributed to hippocampal shape differences between NC and AD. Among these components, several of them (the 1^st^, 8^th^) also contributed to shape differences between NC and MCI (**Table 3**). Interestingly, only the 9^th^, 13^th^, and 17^th^ components showed the shape differences between MCI and AD. Moreover, among these ISOMAP components, the 1^st^ component was highly correlated with the ventricular volume (Pearson correlations: r = 0.98, p<0.01), though it was not the only component contributing to shape differences between NC and MCI, as illustrated in **Figure 3 (a,b)**. However, the 9^th^, 13^th^, and 17^th^ components were not associated with the lateral ventricular volume (p>0.05), suggesting that only local ventricular shapes contributed to the difference between MCI and AD. This can also be seen in **Figure 3 (c,d)**. Unlike the hippocampus, the lateral ventricular shapes did not lead to better classification accuracy rates between NC and AD (61.5%), and between NC and MCI (59%) when compared with the lateral ventricular volumes (see **Table 2**). However, the ventricular shapes achieved markedly better accuracy in distinguishing MCI and AD (60.1%) (**Table 2**).

#### CSF Markers

Student's *t*-tests revealed that all three CSF markers, Aβ42, t-tau, and p-tau, showed statistically significant differences between NC and AD and between NC and MCI (**Table 3**). However, only Aβ42 showed group differences between MCI and AD (**Table 3**). The SVM classification revealed that the CSF markers can distinguish NC and AD at the classification accuracy of 81.4%, higher than those based on the hippocampal or ventricular imaging markers (**Table 2**). Additionally, the CSF markers achieved similar accuracy in distinguishing NC and MCI, and MCI and AD subjects in comparison with hippocampal and ventricular shapes. Interestingly, the CSF markers gave higher sensitivities than the imaging markers (**Table 2**).

#### The combination of the Imaging and CSF Markers

Combining the CSF markers and the volumes of the hippocampus and lateral ventricles as features in the SVM increased the classification accuracies between NC and AD (85.4%) and between NC and MCI (72%) when compared to those achieved using only one type of markers (see **Table 2**). Nevertheless, there was no improvement on the classification accuracy between MCI and AD when compared with that using the CSF markers alone.

Combining the CSF markers and the shapes of the hippocampus and lateral ventricles as features in the SVM increased the classification accuracies between NC and AD (92.2%), between NC and MCI (70.3%), and between MCI and AD (69.6%) when compared to those achieved using only one type of the markers (see Table 2).

Shapes of the two structures with the CSF markers performed better in separating NC and MCI (p<0.001) or NC and AD (p<0.001) when compared to features combining the volumes and CSF markers. However, shapes of the two structures with the CSF markers performed worse in separating NC and MCI when compared to features combining the volumes and CSF markers (p = 0.013).

### Prediction of the MCI Conversion

To predict MCI conversion at a two-year follow-up, we trained an NC and AD classifier using the CSF and imaging markers before applying it to the 25 MCI converters and 86 MCI non-converters. Again, combination of the CSF markers with the hippocampus and lateral ventricles shapes at baseline showed the best prediction (66.7%) and sensitivity (82%) for identifying the MCI converters when compared to individual imaging or CSF markers (**Table 4**).

**Table 4:** The accuracy, sensitivity, and specificity for predicting the MCI converters are listed when the volumes or shapes of the hippocampus (Hp) or the lateral ventricles (LV), or the CSF markers, or their combination were used as features in the classification.

Markers	Accuracy (95% CI)	Sensitivity	Specificity
**Hippocampal volumes and Shapes**			
Hp volumes	54%(53.1%∼54.9%)	54.4%	53.6%
Hp shapes	63%(62.1%∼64%)	74.9%	51.2%
**Lateral Ventricular Volumes and Shapes**			
LV volumes	55.6%(54.7%∼56.5)	63.7%	47.5%
LV shapes	62.7%(61.5%∼63.9%)	67.9%	57.5%
**CSF**			
CSF markers	62.2%(61.3%∼63.1%)	80.4%	44%
**Combination**			
CSF, Hp volumes, LV volumes	59.2%(58.3%∼60%)	81.1%(80.5%∼81.6%)	37.2%(35.6%∼38.9%)
CSF, Hp shapes, LV shapes	66.7%(65.7%∼67.8%)	82%(81.5%∼82.6%)	51.4% (49.4%∼53.3%)

## Discussion

Our study demonstrated the dynamic trajectories of the CSF, hippocampal and lateral ventricular markers in the Alzheimer's pathological cascade using a cross-sectional ADNI sample and also showed the feasibility of predicting future MCI-to-AD conversion using baseline CSF and imaging markers. The CSF markers, including Aβ42, t-tau, and p-tau, distinguished MCI or AD from NC, while only the Aβ42 CSF marker contributed to the differentiation between MCI and AD. The hippocampal shapes performed better than the hippocampal volumes in classifying NC and MCI, NC and AD, as well as MCI and AD. Interestingly, as compared to the ventricular shape, ventricular volume performed better in distinguishing MCI or AD from NC. The ventricular shape, however, showed better accuracy in the classification for MCI and AD. As the CSF and structural markers were complementary, their combination showed great improvement in the classification accuracies at all the stages of AD. Moreover, the combination of these baseline markers also showed high sensitivity but low specificity for predicting MCI conversion to AD during a two-year period.

Our findings supported the conclusion drawn in previous studies [31]; [32], where abnormality of both CSF Aβ42 and neurodegenerative biomarkers, including CSF tau and MRI markers, precedes clinical symptoms; all these markers showed significant differences between NC and MCI groups. However, our findings did not support the hypothesis where CSF Aβ42 reaches a plateau before the appearance of MRI atrophy and cognitive symptoms, and remain static thereafter [7]. In our study, CSF Aβ42 continued to show appreciable power discriminant between MCI and AD. In contrast, CSF tau lost its discriminating power in distinguishing MCI and AD patients, suggesting that CSF Aβ42 reaches its plateau after CSF tau in the Alzheimer's pathological cascade.

MCI and AD patients were not well-separated using hippocampal volume, implying that the overall tissue loss in the hippocampus may not be a good marker for monitoring AD progression. However, we may not conclude that the hippocampus reached its abnormality peak before the late stage of AD, as its local shape variations were significantly associated with progression from MCI to AD. Our results also showed that such local shape markers aided the hippocampal markers in achieving slightly better accuracy than the CSF markers in the prediction for MCI conversion to AD. This was also supported by previous studies, suggesting that MRI markers (e.g. cortical thickness of the medial temporal lobe) correlate well with severity of cognitive impairment and have greater predictive power than the CSF tau [33]. Based on these evidences, we may conclude that the hippocampal shape marker reaches its plateau after CSF tau. However, the order in which the CSF Aβ42 and MRI markers reach their abnormality peaks is still unclear based on our current study.

The volume of the lateral ventricles cannot distinguish MCI and AD patients. Interestingly, the overall expansion of the lateral ventricles showed better performance in identifying MCI or AD from NC when compared with their shapes. This result agrees with previous studies, suggesting that rates of ventricular expansion were significantly different between AD (or MCI) and NC groups [8]. This implies that complicated shape analysis might not always be necessary to provide better structural morphological markers when compared to volumetric analysis. Even for the same structure, its markers can be different at different stages of AD. Again, we may not conclude that the lateral ventricles reached their abnormality peak before the late stage of AD, as their local shape variations were significantly associated with progression from MCI to AD. Likewise, ventricular shape markers slightly outperformed CSF markers in the prediction for MCI conversion to AD, as verified by previous studies [34], wherein clearer correlation was observed for ventricular volumes against worsening cognitive indices, as compared to CSF biomarkers.

Our study showed that the CSF and structural markers are complementary to each other in the AD pathological cascade. This suggests that the CSF markers, (Aβ42, t-tau, and p-tau) with the volumes of the hippocampus and lateral ventricles, is a good combination for distinguishing NC and MCI, while CSF Aβ42 marker with the shape of the hippocampus and lateral ventricles is a good combination for identifying MCI and AD.

As the shapes of the hippocampus and lateral ventricles contributed more to the difference between MCI and AD than their volumes, our study further showed that the combination of the CSF markers and the shapes of the two structures at baseline predicted MCI conversion to AD in the two-year follow up at an improved accuracy of 66.7%. Previous studies [35] achieved similar prediction accuracy (68.5%) for the MCI conversion within a three-year follow up, with suggestions claiming that multiple predictors, including the CSF markers, hippocampal volume, entorhinal cortex thickness, etc. would not perform better than a single predictor. This differs from the conclusion derived from our findings, possibly because structural shape measures contain more complementing features than structural volume measures. The combination of the shapes and CSF markers achieved high classification accuracy (92.2%) between NC and AD, improving by more than 10% over the CSF markers. This result is comparable to those previously reported where CSF, MRI and PET imaging markers were combined [36].

In summary, we conclude that it is feasible to employ a cross-sectional sample to investigate dynamic associations of the CSF and imaging markers with MCI and AD and to predict future MCI conversion to AD. In particular, volumetric information may be good for the early stages of AD while morphological shapes should be considered as markers in the prediction of MCI conversion to AD together with the CSF markers.
